# Maximizing Recovery: The Superiority of Frequent Vacations for Well-Being and Performance

**DOI:** 10.7759/cureus.87569

**Published:** 2025-07-08

**Authors:** Selvaraj Giridharan, Bhuvana Pandiyan

**Affiliations:** 1 Oncology, Tawam Hospital, Al Ain, ARE; 2 Psychiatry, Herefordshire and Worcestershire Health and Care National Health Service (NHS) Trust, Hereford, GBR

**Keywords:** frequency, performance, recovery, vacations, well-being

## Abstract

In contemporary high-pressure work environments, strategic vacation practices are essential for maintaining employee health and productivity. This editorial examines recent research suggesting that frequent short vacations are more effective than infrequent longer breaks in sustaining well-being. We emphasise how regular vacations facilitate recovery and enhance well-being, thereby reducing stress and improving performance. We advocate the integration of frequent breaks into the workplace culture through practical strategies for both individuals and organisations. This analysis calls for researchers, practitioners, and policymakers to reconsider vacation planning and explore the advantages of more frequent vacations.

## Editorial

Introduction

Contemporary workplaces, driven by technological connectivity and global competition, impose escalating demands that erode the boundaries between professional and personal life. This environment generates significant psychobiological strain, as articulated by the effort-recovery theory [1]. This theory posits that sustained work effort depletes physiological and psychological resources, manifesting as elevated cortisol levels, cognitive fatigue, and emotional exhaustion. Without periodic recovery, this strain accumulates, increasing the risk of burnout, reduced productivity, and adverse health outcomes [2]. Vacations, as structured periods of respite, interrupt this cycle, enabling resource restoration, and serving as a critical intervention for sustaining employee well-being. The rising prevalence of work-related stress, evidenced by meta-analytic findings, underscores the urgency of effective recovery strategies such as vacations [3]. However, the optimal structure of vacations, particularly their duration and frequency, requires evidence-based scrutiny to maximise their benefits [4].

Benefits of vacations

Vacations provide substantial psychological, physiological, and cognitive benefits. A meta-analysis conducted by de Bloom et al. synthesised 22 studies that reported significant post-vacation reductions in exhaustion, improvements in mood, and increases in life satisfaction [2]. These findings are consistent with those reported by Grant et al., who reviewed 54 studies and identified physiological improvements, including reduced cortisol levels, enhanced heart rate variability, and better sleep quality [3]. These outcomes reflect mitigation of the physiological burden of chronic stress and positioning vacations as vital health interventions.

Fritz and Sonnentag demonstrated that employees achieving detachment during vacations reported higher energy and lower fatigue, emphasizing the need for complete disconnection [4]. Psychological detachment and mental disengagement from work are the key mechanisms. Additionally, vacations enhance performance and creativity. de Bloom et al. found that restorative activities such as physical exercise, nature immersion, or cultural exploration boost divergent thinking and problem-solving, with effects persisting post-vacation [5]. Positive vacation activities, such as socialising or hobbies, amplify well-being gains, whereas work-related interruptions impede recovery [5].

The duration debate

Although vacations offer numerous benefits, their effects are often constrained by the "fade-out effect." In a study by de Bloom et al., 54 employees were monitored during vacations averaging 23 days, revealing that health and well-being peaked around the eighth day but returned to pre-vacation levels within one week of resuming work [5]. Similarly, Fritz and Sonnentag documented a rapid decline in these benefits, particularly among employees with substantial post-vacation workloads [4]. These findings challenge the notion that longer vacations inherently provide more lasting benefits, indicating that the duration alone is insufficient for sustained recovery.

A recent meta-analysis conducted by Grant et al. offers additional insights into the identification of moderators such as elevated job demands and suboptimal vacation quality, which act as accelerators of the fade-out effect [3]. Employees who return to high-stress environments experience more rapid erosion of benefits, thereby highlighting the interaction between the workplace context and the efficacy of vacations. This finding underscores the necessity of strategies that extend recovery benefits beyond the vacation period.

Frequency as a key factor

Effort-recovery theory's focus on regular recovery is empirically substantiated by evidence indicating that frequent, shorter vacations sustain well-being more effectively than single, extended breaks. Employees who take multiple short vacations annually exhibit higher levels of energy and motivation than those who rely on one prolonged break [5]. Regular opportunities for detachment reduce burnout and enhance job satisfaction. Distributing vacations evenly throughout the year optimises well-being by minimising periods of unchecked stress accumulation [3]. Frequent vacations offer repeated recovery episodes, counteracting the rapid diminishment of benefits, and aligning with the theory's advocacy for consistent resource replenishment.

Evidence-based recommendations

To optimise benefits, individuals are advised to schedule brief vacations every two months to mitigate stress accumulation [1]. It is crucial to completely disconnect from work-related communication to achieve psychological detachment [4]. Participation in restorative activities such as physical exercise or cultural exploration further facilitates recovery. Organisations should implement policies that discourage after-hour communication and provide flexible vacation schedules to promote frequent breaks [3,4]. Wellness programs or vacation planning tools can assist employees in aligning their recovery with workplace objectives.

Conclusion

Vacations are essential for mitigating the psychobiological impacts of contemporary work and providing psychological, physiological, and cognitive advantages. Although the duration of vacations is significant, frequent and shorter vacations are more effective in maintaining well-being through regular recovery. This evidence-based perspective, rooted in effort-recovery theory, advocates strategic vacation planning by organisations and policymakers to prioritise workforce health. Societies should promote vacation policies that emphasise frequency in order to enhance employee well-being and performance.
